# Simultaneous Non-Negative Matrix Factorization for Multiple Large Scale Gene Expression Datasets in Toxicology

**DOI:** 10.1371/journal.pone.0048238

**Published:** 2012-12-14

**Authors:** Clare M. Lee, Manikhandan A. V. Mudaliar, D. R. Haggart, C. Roland Wolf, Gino Miele, J. Keith Vass, Desmond J. Higham, Daniel Crowther

**Affiliations:** 1 Department of Mathematics and Statistics, University of Strathclyde, Glasgow, United Kingdom; 2 College of Medical, Veterinary and Life Sciences, University of Glasgow, Glasgow, United Kingdom; 3 University of Dundee Medical Research Institute, Ninewells Hospital & Medical School, Dundee, United Kingdom; 4 CXR Biosciences Ltd, Dundee, United Kingdom; 5 Epsistem Ltd., Manchester, United Kingdom; 6 Sanofi, Frankfurt am Main, Germany; University of Memphis, United States of America

## Abstract

Non-negative matrix factorization is a useful tool for reducing the dimension of large datasets. This work considers simultaneous non-negative matrix factorization of multiple sources of data. In particular, we perform the first study that involves more than two datasets. We discuss the algorithmic issues required to convert the approach into a practical computational tool and apply the technique to new gene expression data quantifying the molecular changes in four tissue types due to different dosages of an experimental panPPAR agonist in mouse. This study is of interest in toxicology because, whilst PPARs form potential therapeutic targets for diabetes, it is known that they can induce serious side-effects. Our results show that the practical simultaneous non-negative matrix factorization developed here can add value to the data analysis. In particular, we find that factorizing the data as a single object allows us to distinguish between the four tissue types, but does not correctly reproduce the known dosage level groups. Applying our new approach, which treats the four tissue types as providing distinct, but related, datasets, we find that the dosage level groups are respected. The new algorithm then provides separate gene list orderings that can be studied for each tissue type, and compared with the ordering arising from the single factorization. We find that many of our conclusions can be corroborated with known biological behaviour, and others offer new insights into the toxicological effects. Overall, the algorithm shows promise for early detection of toxicity in the drug discovery process.

## Introduction

The aim of this work is to highlight the usefulness of a recently proposed extension to the technique of non-negative matrix factorization (NMF) by demonstrating its promise for early detection of toxicity in the drug discovery process. In particular, we (a) show that any number of related datasets can be treated simultaneously with this approach, (b) deal with practical issues that arise when the algorithm is applied to real datasets, (c) demonstrate its use with a new large scale microrray dataset, and (d) interpret the results from a biological perspective.

### Computational Background

NMF seeks to represent a large complex dataset in terms of smaller factors. The name covers many algorithms. Each approximates a non-negative matrix as the product of two or more smaller non-negative matrices, by attempting to minimise some objective function. Lee and Seung [1] showed that when applying multiplicative non-negative factorization to images of faces, each row/column pair of the factors expresses a recognisable facial feature. These techniques have since been used in many settings to learn parts of the data as well as to factorize and cluster datasets. For example, when applied to text data in [1] the algorithm can differentiate multiple meanings of the same word by context. On microarray data, NMF has been used to find patterns in genes or samples, typically bi-clustering both groups in a similar manner to two-way hierachical clustering [2]–[7]. The review article [8] shows how NMF has also been successful in other areas of computational biology, including molecular pattern discovery, class comparison and biomedical informatics. The new challenge that we address in this work is to apply the NMF methodology to multiple, related, large scale, data sets simultaneously. We use the work of Badea [9], [10], who considered an extension of NMF that deals with two data matrices. Simultaneous NMF is used in [9] to study pancreatic cancer microarray data alongside extra information concerning transcription regulatory factors. In [10] microarray datasets for pancreatic ductal adenocarcinoma and sporadic colon adenocarcinoma are sumiltaneously factorized in order to discover expression patterns common to both data sets. This simultaneous NMF approach readily extends to the case of an arbitrary number of data matrices and here, for what we believe to be the first time, we implement and evaluate the method on more than two. We also consider various practical issues that must be tackled in order to produce a useful computational tool. To minimize the number of algorithmic parameters, make the results straightforward to interpret, and exploit the natural sparsity in the algorithm [9, section 3], we focus on hard clustering. The interesting issue of allowing clusters to overlap in this context is therefore left as future work.

### Biological Background

We analyse gene expression data describing the molecular changes in four tissue types due to different dosages of an experimental pan-peroxisome proliferator-activated receptor (pan-PPAR) agonist PPM-201, provided by Plexxikon. PPARs have attracted great interest as potential therapeutic targets for diabetes [11], but major concerns have arisen due to clinically observed side-effects [12]. Hence, there are compelling reasons for toxicological studies at the gene expression level.

The material is organised as follows. In Section we describe the simultaneous NMF algorithm and outline our approach for using the output to order and cluster a dataset. Section describes the mouse microarray data, and the NMF results that arise when we treat it as a single dataset are given in Section . This is followed in Section by the analysis of the data split into four datasets corresponding to the known tissue types; liver, kidney, heart and skeletal muscle. In Section we compare the gene clusters from Sections and , and Section discusses the results. Conclusions are given in Section .

## Methods

### Algorithms

Given 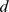 non-negative data matrices 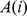 of size 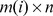 for 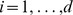, our aim is to simultaneously factorize all matrices so that
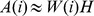
with the additional constraints that 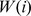 is a non-negative matrix of size 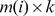 for 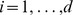, and 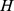 is a non-negative matrix of size 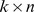. Generalising naturally from the 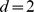 case in [9], we seek to minimise the objective function
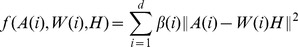
(1)where 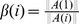. Here 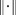 denotes the Frobenius norm. As in [9] the 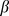 coefficients are designed to give equal weight to the different error terms. Based on the multiplicative update rules developed in [13], an iterative algorithm that attempts to solve the optimisation problem can be derived using a gradient descent method 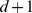 times. This gives us the following sequence of approximations for 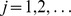, given initial choices 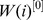 and 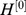,
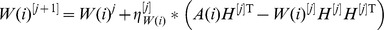


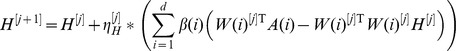
for some small positive matrices 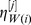, and 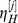, with 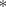 representing element-wise multiplication. The iteration may be motivated through the intuition that when 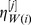 and 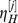 are sufficiently small and positive each of these equations should reduce the objective function. This allows us to set
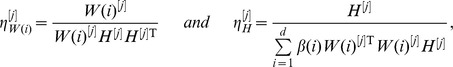
again with the division being performed element-wise. Hence the overall iteration has the form
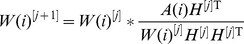


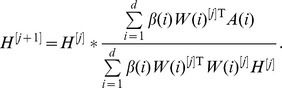



The values in 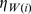 and 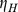 are non-negative due to the constraints on the matrices, however they are not necessarily small. The iteration decreases the objective function (1), so this leads to a locally optimum solution, but we cannot guarantee convergence to a global optimum. In particular, different initial conditions can lead to different factorizations of different quality.

Having iterated up to some stopping criterion and produced the factorizations, we use them to bi-cluster the data. Each sample is assigned to the cluster for which it has the largest value in the gene cluster and vice versa. In reordering the data for easy visualisation we organise the rows and columns by cluster number (assigned arbitrarily) and sort the elements within each cluster from the appropriate sample/gene set, with the largest value at the bottom/right of that cluster. Given that the second factor is common to all the factorizations, it produces a matching ordering of the columns of the data.

Because the result depends on the choice of initial condition, and because the choice of 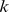 is not automatic, further information is needed in order to specify a practical algorithm. To deal with the lack of uniqueness, we try several initial conditions and pick a realisation that minimises the objective function (1). We then continue until further runs do not significantly alter the results. The objective function value is also one of the criteria we use in order to decide which rank/clustering is the most “appropriate” for the data. By regarding the objective function as a function of 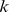, we identify values of 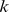 where the decay in the objective function begins to diminish. In addition we also form a consensus matrix as in [3], [14] for the clustering of the objects. This is the average of the connectivity matrices 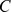 where for each initialisation 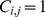 if objects 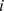 and 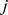 are clustered together and 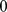 otherwise. So the consensus matrix contains values between 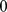 and 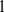 with the 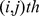 element being the likelihood that objects 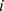 and 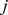 cluster together. The cumulative density of these values is constructed, by summing the appropriate probabilities, and the area under this curve is the second measure we look at when considering choices for 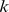. The third measure is the Pearson correlation of the cophenetic distances, as explained in [3].

### Mouse data

We apply these techniques to mouse gene expression data quantifying changes in four different tissue types following administration of different dosages (vehicle, therapeutic and toxic) of an experimental pan-PPAR agonist. The study design and clinical chemistry results are summarised in Table 1. ALT and AST are known markers in rodents for liver toxicity [15] and from this criterion mouse E may be showing a toxic response to PPM201, despite it being administered at a supposedly therapeutic dose level. This conditions our expectation of the gene-expression pattern for mouse E and suggests that it may be similar to the toxic level group III for liver.

**Table 1:** Blood clinical chemistry analysis for each mouse.

Group	Mouse	Dose	ALT	AST	Creatinine	BUN	LDH	CK
ID	ID	(mg/kg b.wt)	(U/L)	(U/L)	( 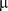 mol/L)	(U/L)	(U/L)	(U/L)
I	A	Vehicle	42	188	12	4	348	484
I	B	Vehicle	41	92	9	6	364	258
I	C	Vehicle	29	75	9	6	278	166
II	D	6	95	441	11	8	1218	4930
II	E	6	692	981	8	7	2126	1130
II	F	6	52	83	9	8	294	152
III	G	20	312	1300	6	8	3172	2544
III	H	20	462	937	8	6	1760	1182
III	I	20	698	1090	6	7	2616	1592

The mice were randomly divided into three groups and treated with either Vehicle or two concentrations of PPM201 (6 or 20 mg/kg body weight). The response to the “therapeutic dose”, 6 mg/kg, was found to vary widely for ALT (alanine aminotransferase), AST (aspartate aminotransferase), LDH (lactate dehydrogenase) and CK (creatine kinase). AST is raised in PPM201 treated animals, with mouse E (6 mg/kg) seeming to be especially raised; AST is known to be variable between animals, but mouse E also shows a higher level of ALT, indicating that there may be a shared mechanism for the two enzymes. Creatinine is decreased in liver and possibly kidney disease; the contrasts observed here are inconclusive. BUN (Blood, Urea and Nitrogen) is raised in kidney disease; results are again inconclusive. Following cardiac infarction LDH is increased after 12 hours, possibly also caused by liver toxicity; mouse E is markedly lower than the other PPM201 treated animals and it may be that its heart muscle profile might be more similar to the untreated mice. CK is, like LDH, increased in myocardial infarction and this supports the LDH findings for mouse E.

Nine wild type mice (strain: C57BL/6J) were randomly divided into three groups; - Group-I, II and III. PPM-201 in the vehicle base was administered daily for 14 days at 6 mg/kg body weight dose rate to each mouse in Group-II and at 20 mg/kg body weight dose rate to each mouse in Group-III while the mice in Group-I received only the vehicle base. On 15th day, the mice were sacrificed to harvest blood, heart, skeletal muscle, liver and kidney tissues for clinical chemistry, microarray and histopathology analysis. In the clinical chemistry analysis, alanine aminotransferase (ALT, U/L), aspartate aminotransferase (AST, U/L), creatinine kinase (CK, U/L), blood urea nitrogen (BUN, mmol/L), creatinine (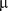
mol/L) and lactate dehydrogenase (LDH, U/L) were measured from the blood of each mouse. Two sections of liver, two sections of kidney, one or two sections of skeletal muscle, and one section of heart were prepared from each mouse, stained with hematoxylin and eosin (H&E), and examined by a veterinary pathologist. Total RNA was isolated from murine tissues using Qiazol-based homogenization and subsequent column-based purification (Qiagen) with on-column DNAse-treatment. DNAse-free RNA was assessed for quality using Agilent Bioanalyser electrophoresis and acceptance criteria of RNA Integrity Number (RIN) greater than seven. 50 ng of total RNA was subsequently utilized as input to cDNA-based amplification and biotin-labelling using single-primer isothermal amplification according to the manufacturer's instructions (Ovation System, NuGEN Technologies). Unlabelled and biotin-labelled cDNA was qualitatively assessed by Agilent Bioanalyser electrophoresis to ensure identical size distributions of all samples pre- and post-fragmentation. Fragmented, biotin-labelled cDNA were hybridized to MOE430 2.0 GeneChip arrays (Affymetrix) with subsequent scanning and feature extraction according to the manufacturer's instructions.

The dataset has been approved by the GEO curators and assigned the accession number GSE31561.

### Ethics Statement

The in vivo procedures undertaken during the course of this study (Ref: CXR0631) were subject to the provisions of the United Kingdom Animals (Scientific Procedures) Act 1986. The study was approved by the CXR Biosciences Local Ethics Committee and complied with all applicable sections of the Act and the associated Codes of Practice for the Housing and Care of Animals used in Scientific Procedures and the Humane Killing of Animals under Schedule 1 to the Act, issued under section of the Act.

## Results

### Single dataset

First, the samples are treated as a single dataset, with thirty six samples and 45037 genes, hence the data matrix 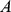 is 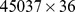. This corresponds to the case where 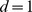 in Section . The factorizations were performed twenty times for each 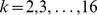, with a consensus matrix formed from the clustering of the samples. All gene clusters associated with this analysis are labelled with a subscript 1, e.g., 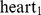.

**Figure 1:**
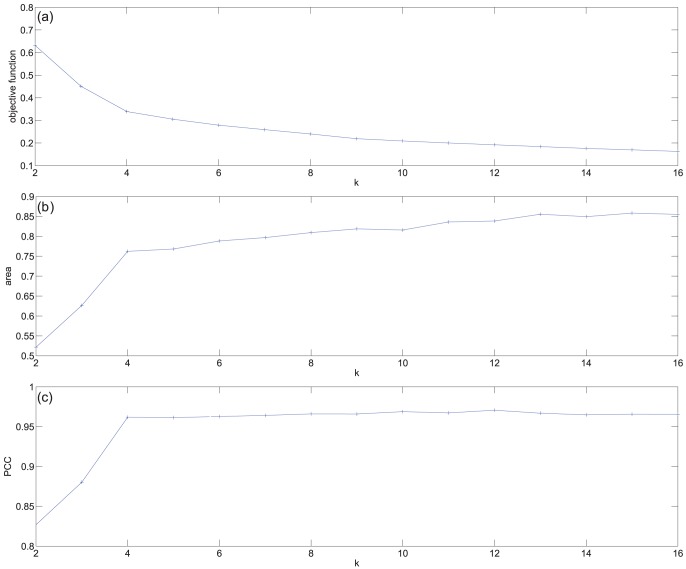
Three measures of the performance versus specified cluster size, 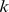
 **, when the data set is factorised as a single entity.** (a) The value of the objective function for 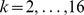. (b) The area under consensus cumulative density, [3], [14]. (c) The cophenetic correlation coefficient, [3].

Figure 1(a) shows the minimum size of the objective function that we observed for each value of 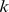. We see that this value decreases monotonically, with a slower rate starting at around 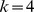. Figure 1(b) shows the area under the cumulative density curves for the same values of 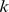. This subfigure clearly points to 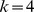, as does subfigure (c) showing the cophenetic correlation.

**Figure 2:**
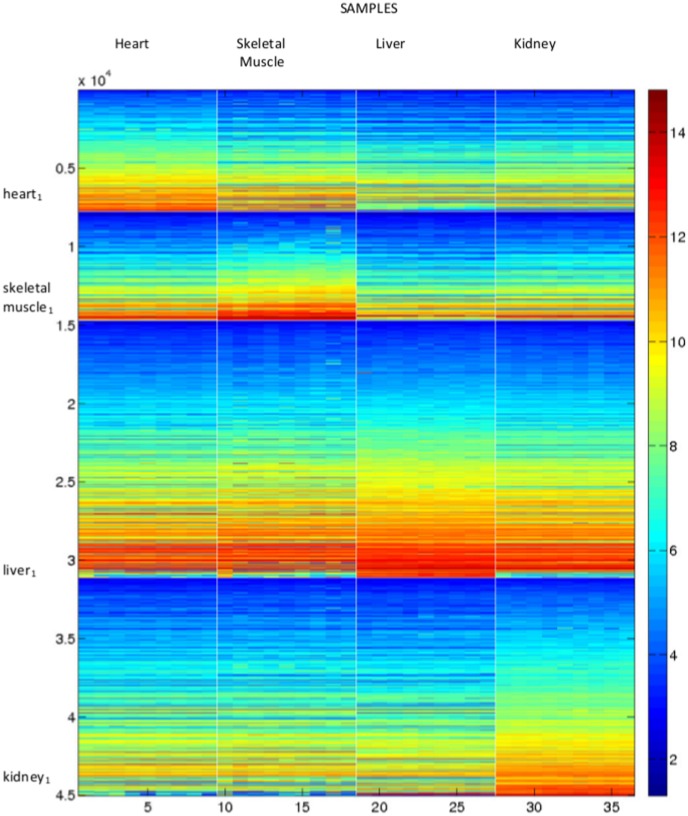
Factorising as a single dataset; reordering using the NMF for 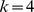
 **.** The columns show the samples and the rows the gene expression for each of the 45037 genes. Genes and samples are organised by cluster number. Elements within each cluster are ordered, with the largest value at the bottom/right. Each tissue is characterised by a group of highly expressed genes; from the top left to bottom right these are heart, skeletal muscle, liver and kidney. For comparison purposes, the characteristic 100 “best” genes in the four columns are names 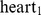, 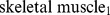, 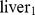 and 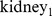.

Based on Figure 1, we conclude that when the data is factorized as a single entity, 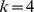 clusters is the most appropriate choice. Reordering the dataset using the ordering for 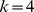 in the manner described in Section gives the images shown in Figure 2. This figure shows the samples in the columns with cluster one at the top. To aid visualisation, the sample clusters are split by white lines, as are the gene clusters. This reordered data matrix shows a distinctive “ramp” effect in the blocks on the diagonal, placing genes that are most influential in identifying each tissue type to the bottom of the block. This figure also shows some of the differences in expression behaviour between the tissue types, particularly for the most influential genes.

Because we know the origin of the samples, we can confirm that the algorithm has put the heart samples in cluster one, the skeletal muscle samples in cluster two, the liver samples in cluster three, and the kidney samples in cluster four. The exact ordering of the samples is shown in Table 2. This table also shows the mouse identification information for each sample, and we see that the mice are not ordered in the same way within each cluster. It is the liver and skeletal muscle samples that most closely respect the dosage levels within the clusters. Both these clusters only have one sample mis-ordered.

**Table 2:** Ordering of the tissue samples after single factorization of rank 4 of the entire dataset.

Cluster	Tissue type	Mouse	Dosage
1	Heart	D	6 mg/kg
1	Heart	B	Vehicle
1	Heart	C	Vehicle
1	Heart	I	20 mg/kg
1	Heart	H	20 mg/kg
1	Heart	A	Vehicle
1	Heart	G	20 mg/kg
1	Heart	E	6 mg/kg
1	Heart	F	6 mg/kg
2	Skeletal Muscle	H	20 mg/kg
2	Skeletal Muscle	D	6 mg/kg
2	Skeletal Muscle	I	20 mg/kg
2	Skeletal Muscle	G	20 mg/kg
2	Skeletal Muscle	F	6 mg/kg
2	Skeletal Muscle	E	6 mg/kg
2	Skeletal Muscle	C	Vehicle
2	Skeletal Muscle	A	Vehicle
2	Skeletal Muscle	B	Vehicle
3	Liver	I	20 mg/kg
3	Liver	H	20 mg/kg
3	Liver	G	20 mg/kg
3	Liver	E	6 mg/kg
3	Liver	A	Vehicle
3	Liver	F	6 mg/kg
3	Liver	D	6 mg/kg
3	Liver	C	Vehicle
3	Liver	B	Vehicle
4	Kidney	G	20 mg/kg
4	Kidney	I	20 mg/kg
4	Kidney	H	20 mg/kg
4	Kidney	E	6 mg/kg
4	Kidney	A	Vehicle
4	Kidney	C	Vehicle
4	Kidney	F	6 mg/kg
4	Kidney	B	Vehicle
4	Kidney	D	6 mg/kg

**Figure 3:**
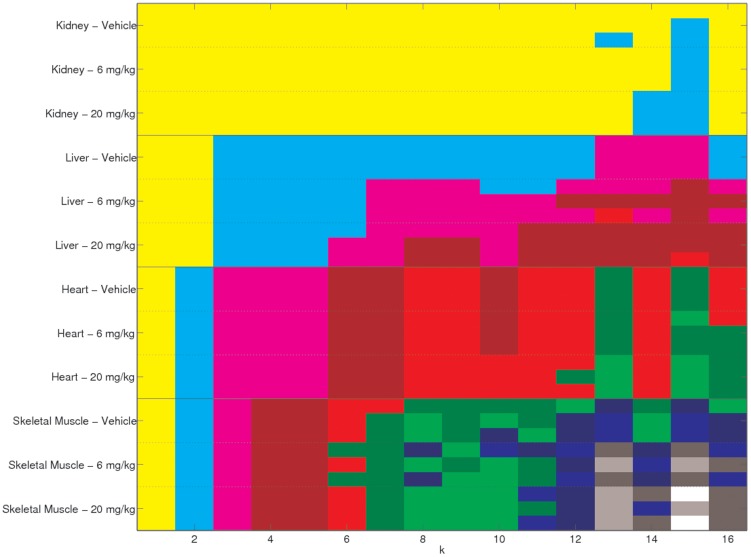
Factorising as a single dataset. The clustering of the mouse samples for 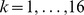. Within each column the samples in the same colour are clustered together. No value of 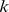 reveals the known tissue/dosage subgroups, or places different tissues in the same cluster.

Given that the factorization has been performed for 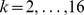 we know what the clustering would be from all these rank factorizations. This information is displayed in Figure 3. Here the rows representing the samples are ordered in tissue then dosage subgroups. For each rank 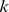, samples with the same colour are assigned to the same cluster. As we have seen before, for 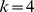 the samples are split into tissue types. The figure shows that this split persists at 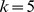 with an empty cluster forming. In fact, for this range of 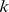 there are at most twelve clusters of samples. We also see from this figure that for no value of 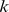 are the twelve tissue/dosage subgroups found.

### Multiple datasets

The test in Section indicates that the basic NMF factorization approach can deliver biologically meaningful results—separating the twelve samples by tissue type. But the failure to order correctly within tissue type according to dosage motivates the use of the multiple dataset generalization introduced in Section , where the four tissue types are treated as separate sources of information across a common set of mice. Intuitively, we would expect to add value to the data analysis by building known biology into the algorithm in this way. In this section, we therefore factorize the four new datasets simultaneously. This is similar to the test in Section in the sense that it produces a single ordering for the mice, but it has the potential to add extra information by providing four different, tissue-level, gene orderings. We thus have 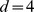 matrices of size 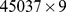. We again performed 20 factorizations, this time for 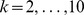 and these have been used to generate a consensus for clustering the mice.

**Figure 4:**
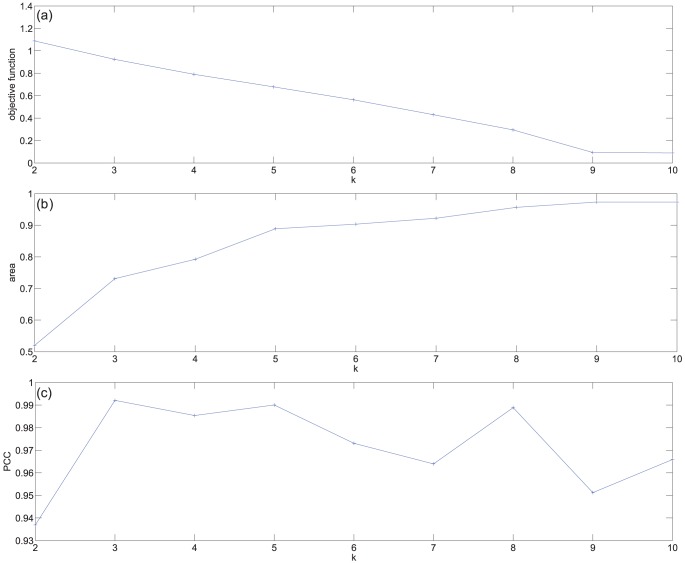
Three measures of the performance versus specified cluster size, 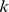
 **, when the four tissue types are factorised separately.** (a) The value of the objective function for 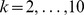. (b) The area under consensus cumulative density function for 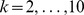, [3], [14]. (c) The cophenetic correlation coefficient, [3].

The objective function and the consensus measurements are shown in Figure 4. The objective function in subfigure (a) does not show much decrease in convergence rate until we get to nine clusters. This is the point where each mouse is put into a cluster on its own. The area under the cumulative density curve in Figure 4(b) suggests using either rank 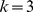, or 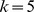 factorizations for the clustering. The correlation coefficients shown in subfigure (c) give the same two values as peaks, as well as 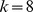, though the 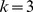 peak is the highest.

**Figure 5:**
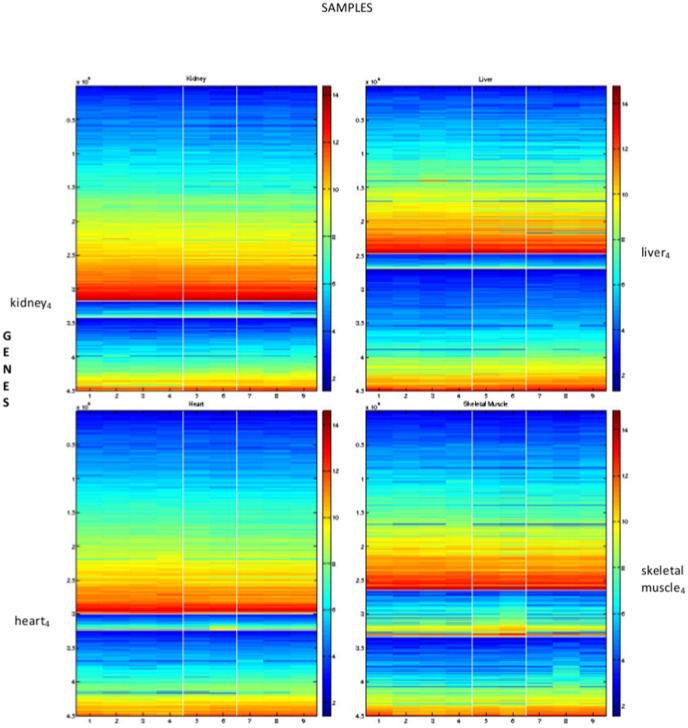
Factorisation of the four separate tissue types using simultaneous NMF with 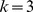
 **.** Top left, kidney; top right, liver; lower left, heart; lower right, skeletal muscle. The four tissue types are treated as separate sources of information across a common set of mice. Genes are therefore ordered differently in each of the four tissues, but the mice ordering is global. The resulting mouse ordering and mouse clusters are detailed in Table 3.

**Figure 6:**
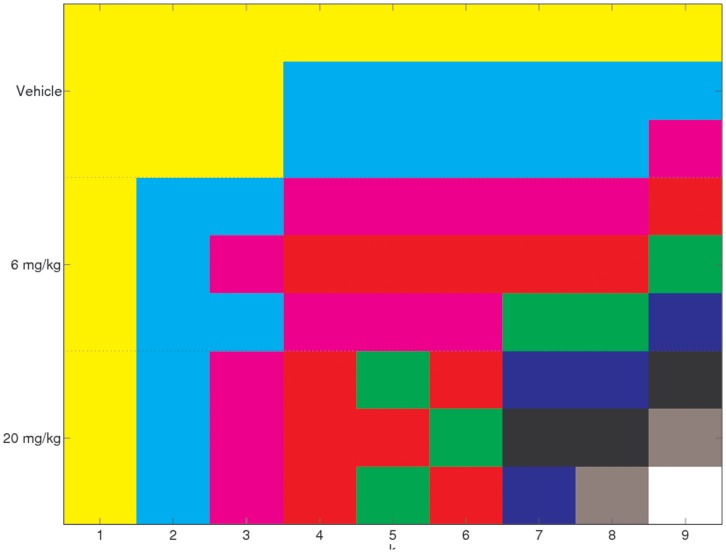
Factorisation of the four separate tissue types simultaneously. The clustering of the mice for 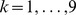; colour indicates cluster number. One “misclassification” is found for several values of 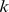. This involves the mouse showing a toxic response to the lower (6 mg/kg) dose of PPAR agonist, as discussed in section .

Given these measurements we consider the four-way simultaneous factorization for 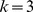 in Figure 5. The reordered datasets are shown separately with the kidney dataset in the top left, the liver dataset in the top right, the heart dataset in the bottom left and the skeletal muscle in the bottom right. The mouse ordering and mouse clusters that arise are shown in Table 3. The four subfigures in Figure 5 also illustrate that the gene clusters are different for each dataset. The three clusters for each tissue in this 4-way factorization are subsequently refered to in the form “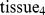, cluster 1,2 or 3. ” Table 3 shows that the simultaneous NMF approach has recovered the known mouse treatments except for one misplacement. Figure 6 shows the clustering for the four-way simultaneous factorizations for 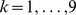. This indicates that this mouse does not cluster with all those of the same dosage for any rank of factorization greater than two, instead it associates with the higher more toxic dosage. This is borne out by the known blood chemistry, as summarised in Table 1; the mouse that is mis-classified exhibits a toxic response and is therefore classified with the mice that received the higher dose.

**Table 3:** Ordering of the tissue samples after a four-way factorization of rank 3.

Cluster	Mouse	Dosage
1	E	6 mg/kg
1	G	20 mg/kg
1	I	20 mg/kg
1	H	20 mg/kg
2	F	6 mg/kg
2	D	6 mg/kg
3	B	Vehicle
3	A	Vehicle
3	C	Vehicle

The mouse clusters when split by tissue type and reordered using the 4-way simultaneous factorization for 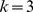.

### Comparing Gene clusters

Our aim now is to test the results from the novel multi-way NMF algorithm used in Section in order to see whether they (a) show consistency and (b) add value to the results in Section from standard NMF. We know that the four simultaneously factorized datasets correspond to the four clusters of samples that were discovered in an unsupervised manner from the single factorization of the full dataset. It could therefore be conjectured that the most influential genes in the first factorization will appear as influential genes in the four-way simultaneous factorization for that dataset, but less so for the other datasets.

Our comparisons involve four reference sets. For illustration, we chose an arbitrary threshold of one hundred; that is, we consider the top one hundred most influential genes from the four clusters in the first factorization shown in Figure 2. For easy reference these sets are referred to using the known tissue type. This means that the genes from cluster one are the 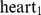 genes, those from cluster two are the 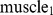 genes, those from cluster three are the 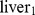 genes and those from cluster four are the 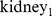 genes. The 4-way factorization shown in Figure 5 identifies differently ordered gene clusters for each tissue, which we will refer to as “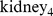, cluster 1,2 or 3, etc. ” Table 4 shows the total number of co-incident genes between the top 100 lists arising from the one-way and four-way factorisations. The table also shows the probability of the two lists having that number of genes in common if the second list were randomly selected; hence these values come from the hypergeometric distribution. We see that the important genes for each tissue type appear significantly highly in the clusters from that tissue's data type. In addition, all the tissue type genes also appear significantly within the reordering of the heart dataset. This link is reciprocated, with the heart genes appearing significantly frequently within the skeletal muscle dataset. Surprisingly, the greatest overlap arose between 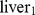 and 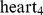 cluster 2. One of these genes, Apoliprotein A1, is being considered as a marker for cardiac toxicity [16].

**Table 4:** Gene cluster comparison for indivdual tissues in the single matrix, “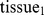,” with the four separate tissue matrices “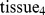.”

		H1		SM1		L1		K1	
Cluster		No.	Probability	No.	Probability	No.	Probability	No.	Probability
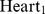	Clust.1	22	2.2188e-38	0	0.8005	0	0.8005	0	0.8005
	Clust.2	1	0.1785	2	0.0195	49	5.444e-108	0	0.8005
	Clust.3	11	4.3469e-16	7	2.8360e-09	1	0.1785	5	3.0037e-06
	total	34	5.4969e-67	9	1.4338e-12	50	6.309e-111	5	3.0037e-06
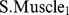	Clust.1	4	7.3075e-05	15	1.1260e-12	8	6.8371e-11	0	0.8005
	Clust.2	0	0.8005	0	0.8005	0	0.8005	0	0.8005
	Clust.3	4	7.3075e-05	14	1.0243e-21	0	0.8005	0	0.8005
	total	8	6.8371e-11	29	1.3672e-54	8	6.8371e-11	0	0.8005
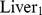	Clust.1	1	0.1785	0	0.8005	13	8.4974e-11	1	0.1785
	Clust.2	0	0.8005	0	0.8005	0	0.8005	0	0.8005
	Clust.3	1	0.1785	2	0.0195	16	1.1336e-25	2	0.0195
	total	2	0.0195	2	0.0195	29	1.3672e-54	3	0.0014
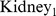	Clust.1	0	0.8005	0	0.8005	1	0.1785	0	0.8005
	Clust.2	2	0.0195	1	0.1785	1	0.1785	0	0.8005
	Clust.3	0	0.8005	0	0.8005	2	0.0195	18	8.9507e-30
	total	2	0.0195	1	0.1785	4	7.3075e-05	18	8.9507e-30

H1, SM1, L1 and K1 are the gene clusters most characteristic for the heart, skeletal muscle, liver and kidney, respectively, in the single (combined) data set, as in Figure 2. Clust.1, 2, or 3 denotes the 100 genes most securely placed within the clusters of the diferently ordered genes in the 4-way factorization shown in Figure 5. The order of the clusters is 1–3, from the top of the figire, for each tissue. We refer to these clusters as “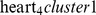,” etc. The overlap of the 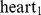 from the one-way factorization to 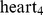 is referred to as 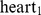 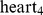 cluster 1.

**Figure 7:**
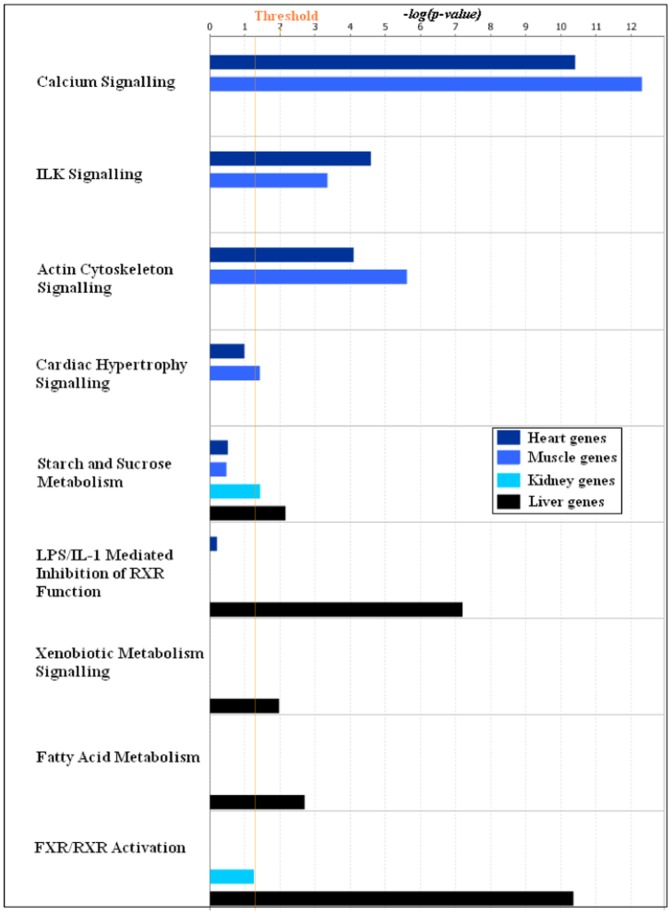
Enrichment of canonical pathways in the four tissue specific gene clusters. The top one hundred most influential probe-sets in the four tissue specific gene clusters obtained in the first factorization were subjected to signalling and metabolic pathways analysis in the IPA software. This graph shows the comparison of canonical pathways enriched in the four tissue specific gene clusters, 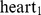, 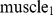, 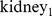 and 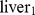. The coloured bars show the significance of the enrichment for a particular pathway in the cluster computed by Fisher's exact test.

**Figure 8:**
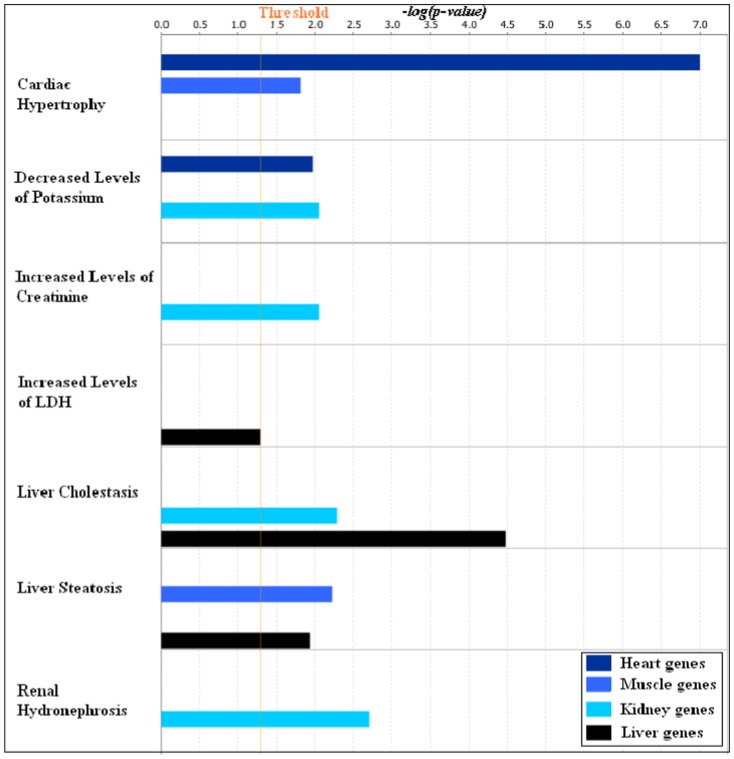
Enrichment of toxicity functions in the four tissue specific gene clusters. The top one hundred most influential probe-sets in the four tissue specific gene clusters obtained in the first factorization were subjected to IPA-Tox analysis in the IPA software. This graph shows the comparison of toxicity functions enriched in the four tissue specific gene clusters. The coloured bars show the significance of the enrichment for a particular toxicity functions in the cluster computed by Fisher's exact test.

We would like to demonstrate the utility of the factorization method by using the gene clusters obtained in our analysis to understand tissue specific effects of the experimental drug, PPM-201. Of course, we are not claiming that this is an exhaustive analysis of the effects of PPM-201. We analysed the gene clusters for pathways enrichment and gene ontology enrichment using DAVID [17] and Ingenuity Pathways Analysis (IPA) [18] tools. Table 5 shows the comparison of KEGG pathways enriched in the four tissue specific top one hundred most influential probe-sets obtained in the first factorization. Pathways enriched in these clusters differ according to the tissue types and can be considered as the pathways that are most perturbed by PPM-201. For example, arrhythmogenic right ventricular cardiomyopathy, hypertrophic cardiomyopathy and dilated cardiomyopathy are enriched in heart, whereas starch and sucrose metabolism, drug metabolism and PPAR signalling pathway are enriched in liver. Similarly, Figure 7 shows the enrichment of canonical pathways in the four tissue specific clusters analysed using IPA. It also shows the tissue specific enrichment of pathways—calcium signalling, integrin linked kinase (ILK) signalling and cardiac hypertrophy signalling are enriched in 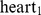 and 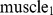 clusters, whereas fatty acid metabolism and farnesoid X receptor (FXR)/retinoid X receptor (RXR) activation are enriched in the 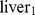 cluster. Analysis of the same sets of genes for enrichment of toxicity functions in the IPA shows, in Figure 8, cardiac hypertrophy in 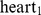 genes, increased level of creatinine and hydronephrosis in 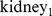 genes, and increased levels of lactate dehydrogenase (LDH) and steatosis in 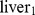 genes.

**Table 5:** Enrichment of KEGG pathways in the four tissue specific gene clusters.

Kegg Pathways	Heart	Muscle	Kidney	Liver
1 mmu05412: Arrhythmogenic right ventricular cardiomyopathy	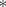	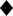		
2 mmu04020: Calcium signalling pathway	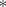	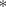		
3 mmu04260: Cardiac muscle contraction	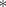	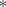		
4 mmu05414: Dilated cardiomyopathy	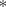	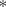		
5 mmu05410: Hypertrophic cardiomyopathy (HCM)	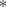	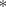		
6 mmu04530: Tight junction	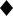	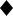		
7 mmu00590: Arachidonic acid metabolism			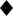	
8 mmu00983: Drug metabolism				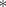
9 mmu04610: Complement and coagulation cascades				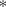
10 mmu00980: Metabolism of xenobiotics by cytochrome P450				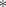
11 mmu03320: PPAR signalling pathway				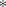
12 mmu00830: Retinol metabolism				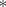
13 mmu00040: Pentose and glucuronate interconversions				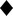
14 mmu00591: Linoleic acid metabolism				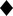
15 mmu00053: Ascorbate and aldarate metabolism				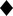
16 mmu00860: Porphyrin and chlorophyll metabolism				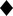
17 mmu00500: Starch and sucrose metabolism				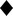
18 mmu00150: Androgen and estrogen metabolism				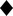
19 mmu00140: Steroid hormone biosynthesis				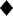

The top one hundred most influential probesets in the four tissue specific gene clusters were analysed using DAVID functional annotation tool. This table shows the comparison of KEGG pathways enriched in the four tissue specific gene clusters. The 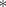 icon indicates a p-value 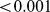 and the 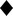 a 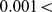
p-value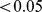 showing the significance of the enrichment.

The common genes between the top one hundred most influential probe-sets in the four tissue specific clusters and the top one hundred most influential probe-sets in the clusters formed by 4-way simultaneous factorization of the split dataset were also analysed for enrichment of pathways, gene ontology and toxicity functions using DAVID and IPA. Tables 6, 7, 8, 9, 10, 11, 12, 13, 14, 15, 16, 17 summarise the results of this analysis, which are discussed further in the next section.

**Table 6:** Enrichment of KEGG pathways in the common genes between the clusters found by the two ways of factorization.

Kegg Pathways	Heart_1_	Heart_1_	Muscle_1_	Liver_1_	Liver_1_	Liver_1_	Liver_1_
	Heart_4_	Muscle_4_	Muscle_4_	Liver_4_	Liver_4_	Heart_4_	Muscle_4_
	clust. 1	clust. 3	clust. 1	clust. 1	clust. 3	clust. 2	clust. 1
1 mmu04020: Calcium signalling pathway			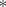				
2 mmu04260: Cardiac muscle contraction	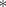	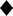					
3 mmu04610: Complement and coagulation cascades						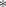	
4 mmu05414: Dilated cardiomyopathy	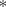	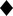					
5 mmu00983: Drug metabolism					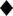		
6 mmu05410: Hypertrophic cardiomyopathy (HCM)	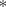	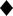					
7 mmu03320: PPAR signalling pathway				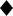		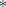	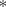
8 mmu04530: Tight junction	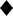		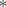				

The probesets common to clusters formed by the 4-way simultaneous factorization and the top one hundred most influential probesets in the four tissue specific clusters were analysed for enrichment of KEGG pathways using DAVID functional annotation tool. Fishers' exact test p-values for pathway enrichment in the clusters are shown graphically in this table. The 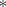 icon indicates a p-value 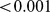 and the 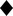 a 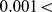
p-value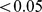.

**Table 7:** Muscle genes present in the calcium signalling pathway.

Sr.	Probeset ID	Gene Symbol	Entrez Gene ID	Entrez Gene Name
1	1427735 a at	ACTA1	11459	Actin, alpha 1, skeletal muscle
2	1419312 at	ATP2A1	11937	ATPase, Ca++ transporting, cardiac muscle, fast twitch 1
3	1422598 at	CASQ1	12372	Calsequestrin 1 (fast-twitch, skeletal muscle)
4	1427520 a at	MYH1	17879	Myosin, heavy chain 1, skeletal muscle, adult
5	1425153 at	MYH2	17882	Myosin, heavy chain 2, skeletal muscle, adult
6	1458368 at	MYH4	17884	Myosin, heavy chain 4, skeletal muscle
7	1452651 a at	MYL1	17901	Myosin, light chain 1, alkali; skeletal, fast
8	1457347 at	RYR1	20190	Ryanodine receptor 1 (skeletal)
9	1440962 at	SLC8A3	110893	Solute carrier family 8, member 3
10	1417464 at	TNNC2	21925	Troponin C type 2 (fast)
11	1416889 at	TNNI2	21953	Troponin I type 2 (skeletal, fast)
12	1450118 a at	TNNT3	21957	Troponin T type 3 (skeletal, fast)
13	1419739 at	TPM2	22004	Tropomyosin 2 (beta)
14	1426144 x at	TRDN	76757	Triadin

Table shows the probe-sets enriched for calcium signalling among the top 100 probe-sets from the 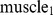 gene cluster.

**Table 8:** Heart genes present in the calcium signalling pathway.

Sr.	Probeset ID	Gene Symbol	Entrez Gene ID	Entrez Gene Name
1	1415927 at	ACTC1	11464	Actin, alpha, cardiac muscle 1
2	1416551 at	ATP2A2	11938	ATPase, Ca++ transporting, cardiac muscle, slow twitch 2
3	1422529 s at	CASQ2	12373	Calsequestrin 2 (cardiac muscle)
4	1448827 s at	MYH6	17888	Myosin, heavy chain 6, cardiac muscle, alpha
5	1448394 at	MYL2	17906	Myosin, light chain 2, regulatory, cardiac, slow
	1427769 x at	MYL3	17897	Myosin, light chain 3, alkali; ventricular, skeletal, slow
7	1421126 at	RYR2	20191	Ryanodine receptor 2 (cardiac)
8	1418370 at	TNNC1	21924	Troponin C type 1 (slow)
9	1422536 at	TNNI3	21954	Troponin I type 3 (cardiac)
10	1440424 at	TNNT2	21956	Troponin T type 2 (cardiac)
11	1423049 a at	TPM1	22003	Tropomyosin 1 (alpha)
12	1451940 x at	TRDN	76757	Triadin

Table shows the probe-sets enriched for calcium signalling among the top 100 probe-sets from the 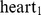 gene cluster.

**Table 9:** Liver genes present in the calcium signalling pathway.

Sr.	Probeset ID	Gene Symbol	Entrez Gene ID	Entrez Gene Name
1	1449817 at	ABCB11	27413	ATP-binding cassette, sub-family B (MDR/TAP), member 11
2	1419393 at	ABCG5	27409	ATP-binding cassette, sub-family G (WHITE), member 5
3	1419232 a at	APOA1	11806	Apolipoprotein A-I
4	1418278 at	APOC3	11814	Apolipoprotein C-III
5	1449309 at	CYP8B1	13124	Cytochrome P450, family 8, subfamily B, polypeptide 1
6	1418190 at	PON1	18979	Paraoxonase 1
7	1450261 a at	SLC10A1	20493	Solute carrier family 10, member 1
8	1449112 at	SLC27A5	26459	Solute carrier family 27, member 5
9	1449394 at	SLCO1B3	28253	Solute carrier organic anion transporter family, member 1B3
10	1424934 at	UGT2B4	71773	UDP glucuronosyltransferase 2 family, polypeptide B4

Table shows the probe-sets enriched for calcium signalling among the top 100 probe-sets from the 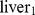 gene cluster.

**Table 10:** 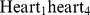
 cluster 1. Common probesets between the top one hundred most influential probesets in the 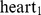 cluster and 20 mg/kg dosage cluster (cluster 1) of the 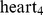 dataset.

Sr.	Probeset ID	Gene Symbol	Entrez Gene Name
1	1415927 at	ACTC1	actin, alpha, cardiac muscle 1
2	1416551 at	ATP2A2	ATPase, Ca++ transporting, cardiac muscle, slow twitch 2
3	1452363 a at	ATP2A2	ATPase, Ca++ transporting, cardiac muscle, slow twitch 2
4	1417607 at	COX6A2	cytochrome c oxidase subunit VIa polypeptide 2
5	1460318 at	CSRP3	cysteine and glycine-rich protein 3 (cardiac LIM protein)
6	1416023 at	FABP3	fatty acid binding protein 3, muscle and heart (mammary-derived growth inhibitor)
7	1453628 s at	LRRC2	leucine rich repeat containing 2
8	1451203 at	MB	myoglobin
9	1418551 at	MYBPC3	myosin binding protein C, cardiac
10	1448554 s at	MYH6	myosin, heavy chain 6, cardiac muscle, alpha
11	1448826 at	MYH6	myosin, heavy chain 6, cardiac muscle, alpha
12	1448394 at	MYL2	myosin, light chain 2, regulatory, cardiac, slow
13	1427768 s at	MYL3	myosin, light chain 3, alkali; ventricular, skeletal, slow
14	1428266 at	MYL3	myosin, light chain 3, alkali; ventricular, skeletal, slow
15	1418769 at	MYOZ2	myozenin 2
16	1450952 at	PLN	phospholamban
17	1423859 a at	PTGDS	prostaglandin D2 synthase 21 kDa (brain)
18	1418370 at	TNNC1	troponin C type 1 (slow)
19	1422536 at	TNNI3	troponin I type 3 (cardiac)
20	1418726 a at	TNNT2	troponin T type 2 (cardiac)
21	1424967 x at	TNNT2	troponin T type 2 (cardiac)
22	1423049 a at	TPM1	tropomyosin 1 (alpha)

**Table 11:** 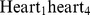
 cluster 3.

Sr.	Probeset ID	Gene Symbol	Entrez Gene Name
1	1422529 s at	CASQ2	calsequestrin 2 (cardiac muscle)
2	1444429 at	LRTM1	leucine-rich repeats and transmembrane domains 1
3	1439101 at	MYLK3	myosin light chain kinase 3
4	1426615 s at	NDRG4	NDRG family member 4
5	1436188 a at	NDRG4	NDRG family member 4
6	1438452 at	NEBL	nebulette
7	1437442 at	PCDH7	protocadherin 7
8	1436277 at	RNF207	ring finger protein 207
9	1423145 a at	TCAP	titin-cap (telethonin)
10	1436833 x at	TTLL1	tubulin tyrosine ligase-like family, member 1
11	1444638 at	TTN	titin

Common probesets between the top one hundred most influential probesets in the 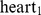 cluster and vehicle dose cluster (cluster 3) of the 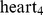 dataset.

**Table 12:** 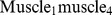
 cluster 1.

Sr.	Probeset ID	Gene Symbol	Entrez Gene Name
1	1427735 a at	ACTA1	actin, alpha 1, skeletal muscle
2	1418677 at	ACTN3	actinin, alpha 3
3	1419312 at	ATP2A1	ATPase, Ca++ transporting, cardiac muscle, fast twitch 1
4	1417614 at	CKM	creatine kinase, muscle
5	1438059 at	CTXN3 (includes EG:629147)	cortexin 3
6	1455736 at	MYBPC2	myosin binding protein C, fast type
7	1427868 x at	MYH1	myosin, heavy chain 1, skeletal muscle, adult
8	1427026 at	MYH4	myosin, heavy chain 4, skeletal muscle
9	1448371 at	MYLPF	myosin light chain, phosphorylatable, fast skeletal muscle
10	1418155 at	MYOT	myotilin
11	1427306 at	RYR1	ryanodine receptor 1 (skeletal)
12	1417464 at	TNNC2	troponin C type 2 (fast)
13	1416889 at	TNNI2	troponin I type 2 (skeletal, fast)
14	1450118 a at	TNNT3	troponin T type 3 (skeletal, fast)
15	1426142 a at	TRDN	triadin

Common probesets between the top one hundred most influential probesets in the 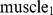 cluster and 20 mg/kg dosage cluster (cluster 1) of the 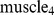 dataset.

**Table 13:** 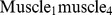
 cluster 3.

Sr.	Probeset ID	Gene Symbol	Entrez Gene Name
1	1453657 at	2310065F04RIK	RIKEN cDNA 2310065F04 gene
2	1434722 at	AMPD1	adenosine monophosphate deaminase 1
3	1460256 at	CA3	carbonic anhydrase III, muscle specific
4	1422598 at	CASQ1	calsequestrin 1 (fast-twitch, skeletal muscle)
5	1439332 at	DDIT4L	DNA-damage-inducible transcript 4-like
6	1427400 at	LBX1	ladybird homeobox 1
7	1419487 at	MYBPH	myosin binding protein H
8	1458368 at	MYH4	myosin, heavy chain 4, skeletal muscle
9	1441111 at	MYLK4	myosin light chain kinase family, member 4
10	1418373 at	PGAM2	phosphoglycerate mutase 2 (muscle)
11	1444480 at	PRKAG3	protein kinase, AMP-activated, gamma 3 non-catalytic subunit
12	1417653 at	PVALB	parvalbumin
13	1422644 at	SH3BGR	SH3 domain binding glutamic acid-rich protein
14	1449206 at	SYPL2	synaptophysin-like 2

Common probesets between the top one hundred most influential probesets in the 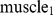 cluster and vehicle dose cluster (cluster 3) of the 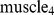 dataset.

**Table 14:** 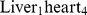
 cluster 2.

Sr.	Probeset ID	Gene Symbol	Entrez Gene Name
1	1449817 at	ABCB11	ATP-binding cassette, sub-family B (MDR/TAP), member 11
2	1425260 at	ALB	albumin
3	1416649 at	AMBP	alpha-1-microglobulin/bikunin precursor
4	1419233 x at	APOA1	apolipoprotein A-I
5	1438840 x at	APOA1	apolipoprotein A-I
6	1455201 x at	APOA1	apolipoprotein A-I
7	1419232 a at	APOA1	apolipoprotein A-I
8	1417950 a at	APOA2	apolipoprotein A-II
9	1417610 at	APOA5	apolipoprotein A-V
10	1417561 at	APOC1	apolipoprotein C-I
11	1418278 at	APOC3	apolipoprotein C-III
12	1418708 at	APOC4	apolipoprotein C-IV
13	1416677 at	APOH	apolipoprotein H (beta-2-glycoprotein I)
14	1424011 at	AQP9	aquaporin 9
15	1419549 at	ARG1	arginase, liver
16	1421944 a at	ASGR1	asialoglycoprotein receptor 1
17	1450624 at	BHMT	betaine–homocysteine S-methyltransferase
18	1451600 s at	CES3	carboxylesterase 3
19	1455540 at	CPS1	carbamoyl-phosphate synthase 1, mitochondrial
20	1418113 at	CYP2D10	cytochrome P450, family 2, subfamily d, polypeptide 10
21	1416913 at	ES1	(includes EG:13884) esterase 1
22	1418897 at	F2	coagulation factor II (thrombin)
23	1417556 at	FABP1	fatty acid binding protein 1, liver
24	1418438 at	FABP2	fatty acid binding protein 2, intestinal
25	1424279 at	FGA	fibrinogen alpha chain
26	1428079 at	FGB	fibrinogen beta chain
27	1416025 at	FGG	fibrinogen gamma chain
28	1426547 at	GC	group-specific component (vitamin D binding protein)
29	1419196 at	HAMP	hepcidin antimicrobial peptide
30	1419197 x at	HAMP	hepcidin antimicrobial peptide
31	1436643 x at	HAMP	hepcidin antimicrobial peptide
32	1425137 a at	HLA-A	major histocompatibility complex, class I, A
33	1448881 at	HP	haptoglobin
34	1423944 at	HPX	hemopexin
35	1434110 x at	LOC100129193	major urinary protein pseudogene
36	1428005 at	MOSC1	MOCO sulphurase C-terminal domain containing 1
37	1417835 at	MUG1	murinoglobulin 1
38	1451054 at	ORM1	orosomucoid 1
39	1418190 at	PON1	paraoxonase 1
40	1417246 at	PZP	pregnancy-zone protein
41	1426225 at	RBP4	retinol binding protein 4, plasma
42	1451513 x at	SERPINA1	serpin peptidase inhibitor, clade A (alpha-1 antiproteinase, antitrypsin), member 1
43	1418282 x at	SERPINA1	serpin peptidase inhibitor, clade A (alpha-1 antiproteinase, antitrypsin), member 1
44	1423866 at	SERPINA3K	serine (or cysteine) peptidase inhibitor, clade A, member 3K
45	1417909 at	SERPINC1	serpin peptidase inhibitor, clade C (antithrombin), member 1
46	1449112 at	SLC27A5	solute carrier family 27 (fatty acid transporter), member 5
47	1449394 at	SLCO1B3	solute carrier organic anion transporter family, member 1B3
48	1419093 at	TDO2	tryptophan 2,3-dioxygenase
49	1422604 at	UOX	urate oxidase, pseudogene

Common probesets between the top one hundred most influential probesets in the 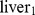 cluster and 6 mg/kg dosage cluster (cluster 2) of the 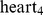 dataset'.

**Table 15:** 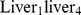
 cluster 1.

Sr.	Probeset ID	Gene Symbol	Entrez Gene Name
1	1425260 at	ALB	albumin
2	1419059 at	APCS	amyloid P component, serum
3	1419232 a at	APOA1	apolipoprotein A-I
4	1419233 x at	APOA1	apolipoprotein A-I
5	1438840 x at	APOA1	apolipoprotein A-I
6	1455201 x at	APOA1	apolipoprotein A-I
7	1417950 a at	APOA2	apolipoprotein A-II
8	1416677 at	APOH	apolipoprotein H (beta-2-glycoprotein I)
9	1419549 at	ARG1	arginase, liver
10	1417556 at	FABP1	fatty acid binding protein 1, liver
11	1428079 at	FGB	fibrinogen beta chain
12	1426547 at	GC	group-specific component (vitamin D binding protein)
13	1448881 at	HP	haptoglobin

Common probesets between the top one hundred most influential probesets in the 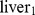 cluster cluster and 20 mg/kg dosage cluster (cluster 1) of the 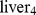 dataset.

**Table 16:** 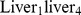
 cluster 3.

Sr.	Probeset ID	Gene Symbol	Entrez Gene Name
1	1428981 at	2810007J24RIK	RIKEN cDNA 2810007J24 gene
2	1449817 at	ABCB11	ATP-binding cassette, sub-family B (MDR/TAP), member 11
3	1417085 at	AKR1C4	aldo-keto reductase family 1, member C4 (chlordecone reductase; 3-alpha hydroxysteroid dehydrogenase, type I; dihydrodiol dehydrogenase 4)
4	1451600 s at	CES3	carboxylesterase 3
5	1449242 s at	HRG	histidine-rich glycoprotein
6	1431808 a at	ITIH4	inter-alpha (globulin) inhibitor H4 (plasma Kallikrein-sensitive glycoprotein)
7	1434110 x at	LOC100129193	major urinary protein pseudogene
8	1420465 s at	LOC100129193	major urinary protein pseudogene
9	1426154 s at	LOC100129193	major urinary protein pseudogene
10	1420525 a at	OTC	ornithine carbamoyltransferase
11	1436615 a at	OTC	ornithine carbamoyltransferase
12	1448680 at	SERPINA1	serpin peptidase inhibitor, clade A (alpha-1 antiproteinase, antitrypsin), member 1
13	1448506 at	SERPINA6	serpin peptidase inhibitor, clade A (alpha-1 antiproteinase, antitrypsin), member 6
14	1449394 at	SLCO1B3	solute carrier organic anion transporter family, member 1B3
15	1424934 at	UGT2B4	UDP glucuronosyltransferase 2 family, polypeptide B4
16	1422604 at	UOX	urate oxidase, pseudogene

Common probesets between the top one hundred most influential probesets in the 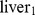 cluster and vehicle dose cluster (cluster 3) of the 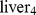 dataset.

**Table 17:** 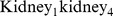
 cluster 3.

Sr.	Probeset ID	Gene Symbol	Entrez Gene Name
1	1456190 a at	ACSM2A	acyl-CoA synthetase medium-chain family member 2A
2	1427223 a at	ACSM2A	acyl-CoA synthetase medium-chain family member 2A
3	1425207 at	BC026439	cDNA sequence BC026439
4	1424713 at	CALML4	calmodulin-like 4
5	1424592 a at	DNASE1	deoxyribonuclease I
6	1448485 at	GGT1	gamma-glutamyltransferase 1
7	1460233 at	GUCA2B	guanylate cyclase activator 2B (uroguanylin)
8	1415969 s at	KAP	kidney androgen regulated protein
9	1415968 a at	KAP	kidney androgen regulated protein
10	1435094 at	KCNJ16	potassium inwardly-rectifying channel, subfamily J, member 16
11	1450719 at	MEP1A	meprin A, alpha (PABA peptide hydrolase)
12	1418923 at	SLC17A3	solute carrier family 17 (sodium phosphate), member 3
13	1417072 at	SLC22A6	solute carrier family 22 (organic anion transporter), member 6
14	1423279 at	SLC34A1	solute carrier family 34 (sodium phosphate), member 1
15	1425606 at	SLC5A8	solute carrier family 5 (iodide transporter), member 8
16	1449301 at	SLC7A13	solute carrier family 7, (cationic amino acid transporter, y+ system) member 13
17	1435064 a at	TMEM27	transmembrane protein 27
18	1423397 at	UGT2B17	UDP glucuronosyltransferase 2 family, polypeptide B17

Common probesets between the top one hundred most influential probesets in the 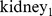 cluster and vehicle dose cluster (cluster 3) of the 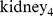 dataset.

## Discussion

**Figure 9:**
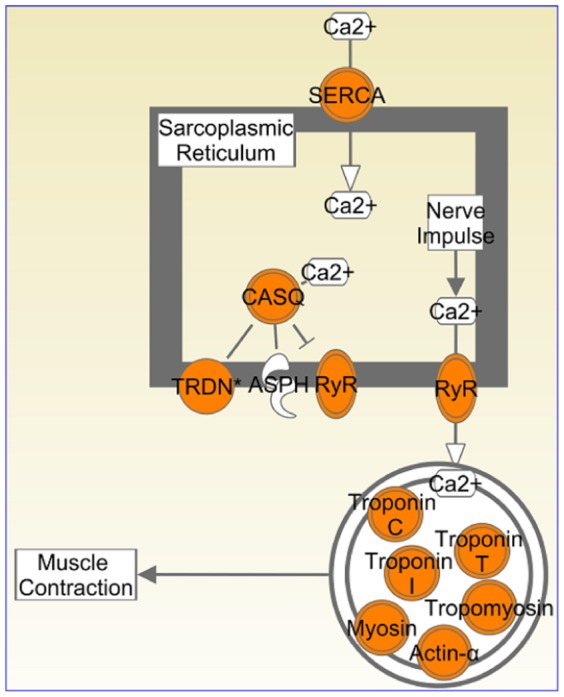
Heart and muscle genes enriched in calcium signalling – muscle contraction pathway. IPA analysis of the top 100 probe-sets from heart and muscle gene clusters (Figure 7) showed the enrichment of calcium signalling pathway. In this figure, we have highlighted the genes present in this pathway in orange. Though this pathway is generalised for skeletal muscle contraction and cardiac muscle contraction, they differ in the members of the same gene family. The heart and muscle genes present in this pathway are given in Tables 7 and 8. Pathway diagram was drawn using Path Designer function of IPA [18].

**Figure 10:**
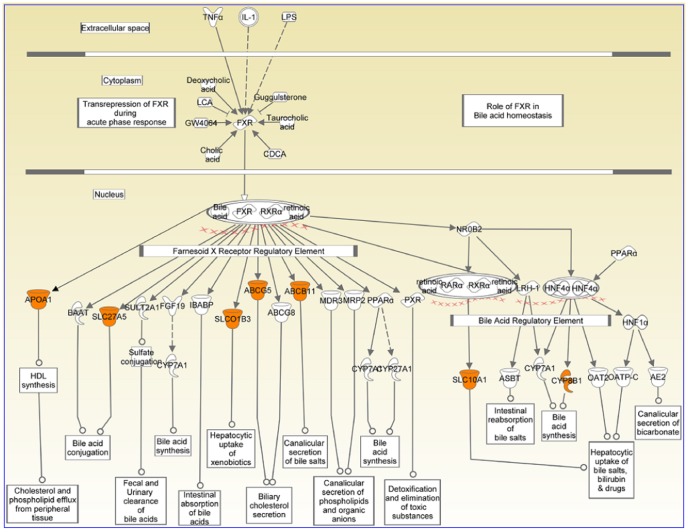
Liver genes enriched in FXR/RXR activation pathway IPA analysis of the top 100 probe-sets from the 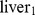
 **cluster (** **Figure 7** **) showed the enrichment of FXR/RXR activation pathway.** The genes present in this pathway are highlighted in orange. The liver genes present in the pathway are given in Table 9. Pathway diagram was drawn using Path Designer function of IPA [18].

The factorization and reordering of the dataset as a whole set (Figure 2 and Table 2) successfully clustered samples from the same tissue and further investigation showed that it simultaneously identified genes with a known relevance to those tissues. It was therefore reasonable to study the genes that were responsible for this differentiation. In the one-way clustering, the top 100 probe-sets from each of the four tissue specific clusters show remarkable coherence for tissue specific pathways. The calcium signalling pathway is highly enriched in both 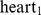 and 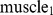 clusters; these genes are linked to muscle contraction function. Muscle contraction is the prime function of cardiac and skeletal muscles. A deeper look at the probe-sets (Tables 7 and 8) from the heart and skeletal muscle clusters shows a successful identification of differences in the tissue types for this pathway; see Figure 9. MYH1, MYH2, MYH4 and MYL1 of the myosin family, which are specific to skeletal muscle, are found in the 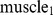 cluster while cardiac muscle specific myosin family members MYH6, MYL2 and MYL3 are found in the 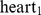 cluster [19]. This pattern is also true for troponin, calsequestrin, ryanodine and actin family members [20]–[25] (Tables 7 and 8). FXR/RXR activation pathway genes are significantly enriched in 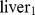 cluster (Figure 7) with most of the enriched genes present in the bile acid synthesis and regulation (Figure 10) pathway, which is one of the core functions of liver [26]–[28]. FXR/RXR activation is also found in the 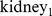 cluster, albeit with moderate significance; FBP1 and HNF4A are the two genes present in this pathway and they may be involved in gluconeogenesis in kidney [29].

Splitting the dataset into four on the basis of tissue types and simultaneous non-negative factorization of them gave us the added reassurance of clustering the samples according to the dosage groups (Figure 5 and Table 3). The clustering of one mouse (Mouse E) from the lower dosage group (Group-II) with the higher dosage group (Group-III) can be explained by the higher PPM201 drug sensitivity of that mouse, indicated by the elevated levels of the toxocology markers ALT, AST, LDH and CK, compared with the rest of its group (Table 1). Comparisons of top probe-sets in tissue specific clusters with dosage specific clusters also show very high overlap of tissue specific genes in the four tissue types. 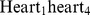 cluster1 has 22 probe-sets that are common between the top 100 probe-sets of 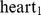 cluster and 20 mg/kg dosage cluster of 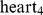 dataset, and are highly enriched for cardiac muscle contraction and hypertrophic cardiomyopathy pathways (Table 6). ACTC1, ATP2A2, MYH6, MYL2, MYL3, TNNC1, TNNI3, TNNT2 and TPM1 are the genes enriched for these two pathways and shared between these two clusters. However, 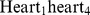 cluster 3, with 11 probe-sets in common between the top 100 probe-sets of 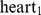 cluster and vehicle dose cluster of 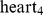 dataset, does not show enrichment for cardiac muscle contraction and hypertrophic cardiomyopathy pathways. From this we may assume that perturbation of cardiac muscle contraction and hypertrophic cardiomyopathy pathways by 20 mg/kg dosage may indicate toxic responses. We also see a similar pattern in skeletal muscle. Between the top 100 probe-sets of 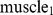 cluster and 20 mg/kg dosage cluster of 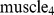, and between the top 100 probe-sets of 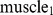 and vehicle dose cluster of 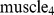, 15 and 14 probe-sets were in common and are named as 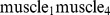 cluster 1 and 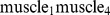 cluster 3, respectively. The calcium signalling–skeletal muscle contraction pathway is enriched in 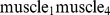 cluster 1 with the presence of ACTA1, ATP2A1, MYH1, MYH4, RYR1, TNNC2, TNNI2, TNNT3 and TRDN genes, whereas 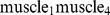 cluster 3 does not show any significant enrichment for signalling or metabolic pathways.

**Figure 11:**
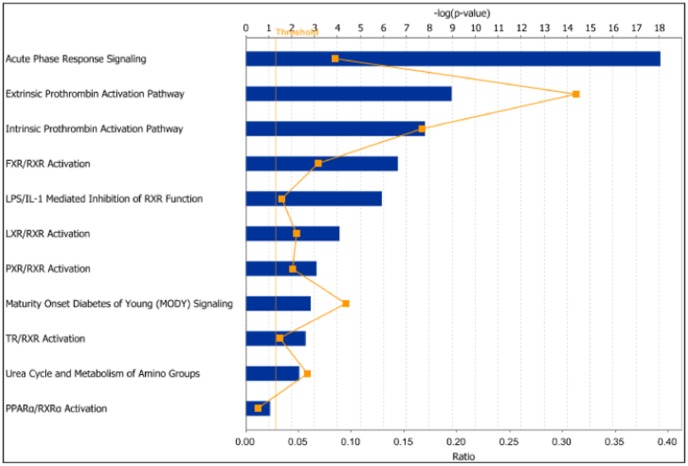
Enrichment of canonical pathways in the liver heart gene cluster no. 2. This gene cluster has 49 common probe-sets between the top one hundred most influential probe-sets in the liver gene cluster and top one hundred probe-sets in cluster number 2 (6 mg/kg dose rate) of the heart dataset reordered by 4-way simultaneous factorization. Canonical pathways enrichment for these 49 probe-sets analysed using the IPA software is shown in this figure. The length of the bars shows the Fisher's exact test p-value for enrichment for a particular pathway in the cluster.

**Figure 12:**
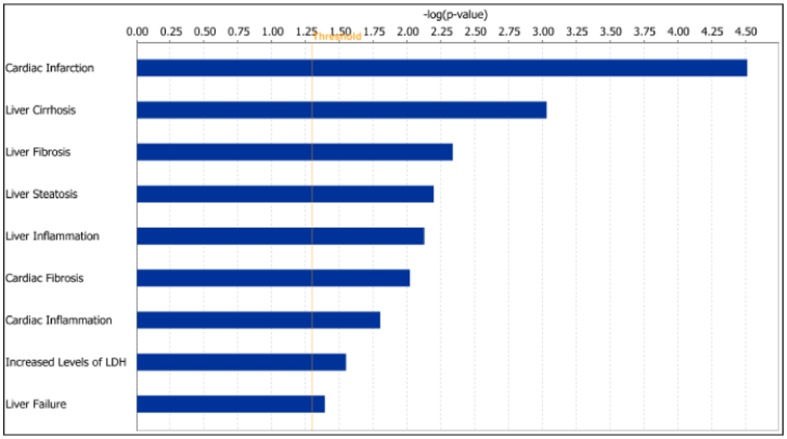
Enrichment of toxicity functions in 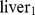
 **** 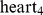 **cluster 2.** This gene cluster has 49 common probe-sets between the top one hundred most influential probe-sets in 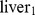 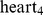 cluster 2 (6 mg/kg dose rate). Toxicity functions enrichment for these 49 probe-sets analysed using the IPA software is shown in this figure. The length of the bars shows the Fisher's exact test p-value for enrichment for a particular pathway in the cluster.

Interestingly, 49 probe-sets in the 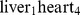 cluster 2 are common between the top 100 probe-sets of 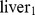 cluster cluster and 6 mg/kg dosage cluster of 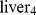 and highly enriched for acute phase response signalling, prothrombin activation and FXR/RXR activation pathways with the presence of ALB, ABCB11, AMBP, APOA1, APOA2, APOC3, APOH, F2, FGA, FGB, FGG, HAMP, HP, HPX, ORM1, PON1, RBP4, SERPINA1, SERPINC1, SLC27A5 and SLCO1B3 genes (Figure 11). This suggests alterations in lipid metabolism in liver along with tissue injury in heart induced by PPM-201 at 6 mg/kg dosage [30]–[33], which becomes more plausible when we look at the genes in 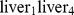 cluster 1 that are common between the top 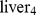 genes and 20 mg/kg dosage cluster of 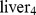 dataset. Enrichment of toxicity functions in 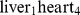 cluster 2 using IPA shows increased level of LDH as one of the toxicity functions (Figure 12) which has been validated with the increased level of LDH in the clinical chemistry results.

## Conclusions

We have demonstrated that multi-way simultaneous nonnegative matrix factorization can be usefully applied to the case of multiple datasets—here, for what we believe to be the first time, more than two large scale matrices were treated. The results were shown to be consistent with, and to add value to, standard nonnegative matrix factorization of the whole dataset.

In summarizing our biological findings, we first note that the roles of the three different isoforms of PPARs - PPAR-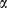, PPAR-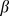 (also known as PPAR-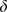) and PPAR-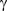 in metabolism and their difference in expression in different tissues and different species are well known [34]–[36]. In mouse, PPAR-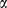 is highly expressed in liver and to a lesser degree in kidney, heart and skeletal muscle; PPAR-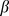 is expressed in many tissues but peaks in kidney, heart and intestine whereas PPAR-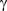 is mostly expressed in adipose tissue [34], [37]. Pan-PPAR agonists activate two or all of the pan-PPAR isoforms and differ in their pharmacological actions. Factorisation of the dataset after splitting it on the tissue basis appears to be beneficial in identifying tissue specific and dosage effects of the experimental pan-PPAR agonist PPM-201 in this study. This approach could be useful in understanding molecular mechanisms and identifying potential tissue specific toxicological effects before they are apparent in histopathology studies. In this study, histopathology examination of heart did not show any defect though our method of gene expression analysis could identify enrichment of acute phase response signalling genes in heart that may point towards building up of toxic responses in heart. Given the fact that many PPAR agonist drugs have been shown to cause cardiac toxicity on prolonged usage and FDA's requirement of one year toxicity study for PPAR agonist drugs, our results show promising early detection of toxicity in the drug discovery process.

Overall, our aim here is to establish a proof of principle for the approach of simultaneously analysing multiple, related large datasets. We therefore focused on a dataset where clear-cut validation is possible. However, we note that the technique is very general, and therefore opens up many new opportunities in data-driven computational biology. In particular, it can be applied to heterogeneous sources of data; for example, generated by different laboratories or experimental methodologies. We are currently pursuing this approach in the study of colon cancer.
